# Biobanking of patient and patient-derived xenograft ovarian tumour tissue: efficient preservation with low and high fetal calf serum based methods

**DOI:** 10.1038/srep14495

**Published:** 2015-10-06

**Authors:** Nicolette G. Alkema, Tushar Tomar, Evelien W. Duiker, Gert Jan Meersma, Harry Klip, Ate G. J. van der Zee, G. Bea A. Wisman, Steven de Jong

**Affiliations:** 1 University of Groningen, University Medical Centre Groningen, Department of Gynaecologic Oncology, Groningen, The Netherlands; 2 University of Groningen, University Medical Centre Groningen, Department of Pathology and Medical Biology, Groningen, The Netherlands; 3 University of Groningen, University Medical Centre Groningen, Department of Medical Oncology, Cancer Research Centre Groningen, Groningen, The Netherlands

## Abstract

Using patient-derived xenografts (PDXs) for preclinical cancer research demands proper storage of tumour material to facilitate logistics and to reduce the number of animals needed. We successfully established 45 subcutaneous ovarian cancer PDXs, reflecting all histological subtypes, with an overall take rate of 68%. Corresponding cells from mouse replaced human tumour stromal and endothelial cells in second generation PDXs as demonstrated with mouse-specific vimentin and CD31 immunohistochemical staining. For biobanking purposes two cryopreservation methods, a fetal calf serum (FCS)-based (95%v/v) “FCS/DMSO” protocol and a low serum-based (10%v/v) “vitrification” protocol were tested. After primary cryopreservation, tumour take rates were 38% and 67% using either the vitrification or FCS/DMSO-based cryopreservation protocol, respectively. Cryopreserved tumour tissue of established PDXs achieved take rates of 67% and 94%, respectively compared to 91% using fresh PDX tumour tissue. Genotyping analysis showed that no changes in copy number alterations were introduced by any of the biobanking methods. Our results indicate that both protocols can be used for biobanking of ovarian tumour and PDX tissues. However, FCS/DMSO-based cryopreservation is more successful. Moreover, primary engraftment of fresh patient-derived tumours in mice followed by freezing tissue of successfully established PDXs is the preferred way of efficient ovarian cancer PDX biobanking.

Intrinsic and acquired resistance to platinum-based chemotherapy is a major obstacle in the treatment of patients with epithelial ovarian cancer and more representative experimental models are an important step in improving bench-to-bedside transition^1^. Xenografts derived from cell lines have been widely used as a preclinical drug testing platform, however with accumulating scepticism about their clinical predictive value. The development of patient-derived xenograft (PDX) models has been of interest for decades, including ovarian cancer based PDX models^2^,^3^,^4^,^5^. Only recently these models are being fully appreciated for their possible application in pre-clinical drug testing^6^. PDX tumours not only accurately phenocopy the patient’s tumour from which they are derived at histological level^7^, but also at expression level^8^,^9^,^10^, mutational status^11^ and they preserve copy number variants for multiple generations^12^,^13^. Furthermore, PDXs resemble their corresponding patient tumour in terms of biological behaviour with engraftment rates directly correlated with poorer overall survival and increased metastatic potential^3^,^7^,^14^. Notably, response rates of implanted tumour grafts against various conventional agents as well as investigational drugs have been reported to correlate with responses of patients^15^,^16^,^17^,^18^.

One of the major issues with PDX models is the varying engraftment rate, with reported latency times varying between 1 to 10 months^19^. Therefore, development of a PDX model will take considerable time. Furthermore, it is recommended to maintain the PDX model at a relatively low passage number (<10) to conserve genetic and histological integrity of the original tumour^11^. Besides this, the histological subtypes of ovarian cancer are not equally represented in ovarian cancer patients, with high-grade serous ovarian cancer being the predominant subtype among the other three main subtypes, i.e. clear cell, mucinous, and endometrioid, and developing a representative model for drug testing will require a large cohort of histological identical patients. Considering all the aforementioned factors, the need for reliable and suitable preservation methods for ovarian cancer PDX biobanking is indispensable. Such a biobank would ideally serve to store patient material and propagated PDX material for reimplantion when required. In general, freezing protocols are based upon the usage of fetal calf serum (FCS) (90–95%) combined with DMSO (5–10%), but not much is known about take rate and growth using this method. In this study, we present our panel of ovarian cancer PDXs together with two methods to preserve human ovarian tumour tissues, derived from both patients as well as from their corresponding established PDX model. We have tested a 95% FCS/5% DMSO protocol as well as a vitrification-based protocol using 10% FCS and stepwise increasing concentrations of DMSO, propanediol, polyvinylpyrrolidone and ethylene glycol^20^. We have carefully analysed both methods in terms of take- and growth rate and resemblance to the parental patient tumour using immunohistochemistry and copy number alterations.

## Results

### Patient characteristics and establishment of PDX model

Between April 2011 and December 2014, tumour tissue from 66 advanced stage (III/IV) ovarian cancer patients was implanted in mice. From these, 45 PDXs were successfully established (take rate 68%) (Table 1 and Supplementary Table S1). Tumours were collected either during primary surgery (n = 28), interval debulking (n = 12), or at relapse (n = 5), and were successfully engrafted in mice, as well as stored using vitrification and/or FCS/DMSO method. The tumours that did not engraft (n = 21) were obtained from primary surgery in 7 cases and from interval debulking in 14 cases, suggesting that primary engraftment is more successful when tumour tissue is not exposed to chemotherapy compared to tumour tissue obtained from interval debulking (p < 0.003). Furthermore, there was a significantly lower amount of vital tumour cells in pieces that did not engraft compared to pieces that successfully engrafted (p < 0.01) (Supplementary Fig. S1). Moreover, in 60% of the tumour samples that did not successfully engraft in mice, vital tumour cell percentage was below 10%. However, in the established PDX group, only 13% of the tumour samples contained less than 10% viable tumour cells.

**Figure 1:**
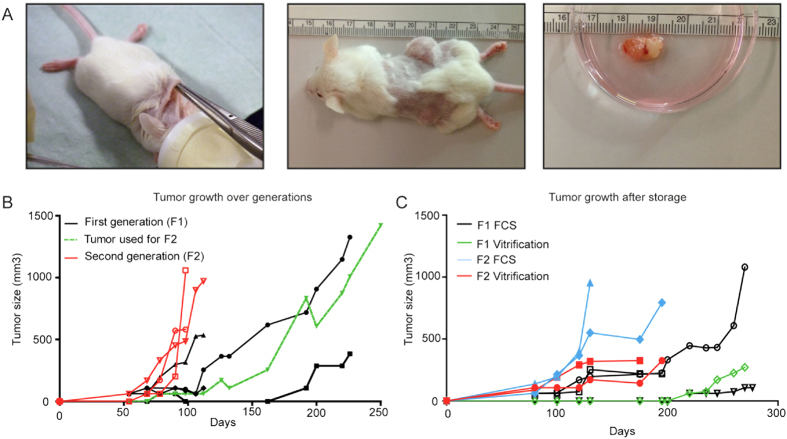
Establishment of the ovarian cancer PDX model. (**A**) Making a single cut in the neck, two pieces were subcutaneously transferred to and implanted on either side of the flank of 6–12 weeks old female NOD.Cg-Prkdcscid Il2rgtm1Wjl/SzJ mice. Tumours were measured once or twice a week and after reaching appropriate size, tumours were harvested for either direct propagation into a further generation or for storage. (**B**) Tumour growth of fresh implanted tumour tissue from patient 56 and further propagation of the tumour (green line) into the second generation (red lines). (**C**) Tumour growth of stored and subsequently thawed and re-implanted tumour tissue from patient 56. Tumour tissue was either directly frozen after patients primary surgery (F1) using either the vitrification (green line) or FCS/DMSO (black line) protocol. After establishment of a PDX, tumour tissue was harvested from the mouse (F2) and frozen using either the vitrification (red line) or FCS/DMSO (blue line) protocol.

For proper cross-verification of ovarian cancer patients, an experienced gynaecologic-oncological pathologist reviewed histological slides of the tumour to reconfirm the diagnosis. Pathological examination diagnosed high-grade serous adenocarcinoma for 31 cases, endometrioid carcinoma for 7 cases, clear cell carcinoma for 3 cases, mucinous carcinoma in 1 case and a mixed phenotype tumour for 3 cases (Table 1). Median latency time, defined as time from implantation till first tumour growth was observed, was 43 days but varied between histology subtypes (Table 1). After reaching a size of at least 1 cm^3^, tumours were harvested and serially transplanted in mice to establish further generations, as well as stored using the vitrification and/or FCS/DMSO method (Fig. 1A). After successful establishment in first generation (F1), all tumours showed successful engraftment in further generations.

### Systematic analysis of biobanking methods

To investigate the preservation methods, we analysed 8 ovarian tumours in more depth. In 8 cases, 6 serous high-grade adenocarcinomas (PDX numbers 30,36,37,56,67 and 84), 1 endometrioid adenocarcinoma (PDX number 157) and 1 mixed ovarian tumour consisting of a teratoma with a borderline mucinous component (PDX number 61), we either thawed primary tumour tissue and/or preserved harvested tumours from successfully established PDXs to test both preservation methods. Overall take rate for freshly transplanted tumour pieces in the F1 generation was 27/44 (61%), with tumours from patient numbers 36 and 67 showing growth only for 1 out of 6 implanted tumour pieces (Table 2). For patient 36 this might be explained by a low amount of vital tumour cells in the primary tumour (Supplementary Fig. S3). Harvested tumours were cut into 6 to 10 pieces for further propagation and storage. PDX tumour tissue from F1, representing each of the 8 patients, was transplanted in mice as F2 generation using a total of 34 pieces (4–5 pieces per harvested tumour), of which 31 grew (91%) (Supplementary Table S2).

In addition, patient tumour specimens were directly frozen using either the vitrification protocol (n = 5 patients) or the FCS/DMSO protocol (n = 3 patients) (Supplementary Table S2). Using the vitrification method on primary material 4 out of 5 patient samples engrafted successfully, however the overall take rate of implanted tumour pieces was 38% (8/21 tumour pieces). The FCS/DMSO method was successful for 2 out of 3 patient tumours and showed an overall take rate of tumour pieces of 67% (6/9 tumour pieces) (Table 2).

After successful expansion of fresh patient tumours as first generation in mice, tumours were also harvested and stored using the vitrification and/or the FCS/DMSO method. With both methods, subsequent establishment of F2 generations was successful in all cases. Overall tumour piece take rates, however, were significantly better using the FCS/DMSO protocol when compared to the vitrification protocol, achieving take rates of 32/34 (94%) and 16/24 (67%), respectively (p = 0.011) (Table 2 and Supplementary Table S2).

Latency period till growth varied between 10 to 270 days for primary tumours and 7 to 104 days in F2 generations (Supplementary Table S2). For vitrified primary tumours latency time period of F1 generation varied from 70 to 320 days, whereas with FCS/DMSO it varied between 18 to 220 days. No statistical differences in latency time were found between fresh and/or stored tissues probably due to the wide spread of latency time among PDXs. Frozen tissue samples, harvested from already established PDXs in F1 showed a latency period in F2 ranging from 35 to 155 days for vitrification and 10 to 115 days for FCS/DMSO (Supplementary Table S2).

Figure 1B,C show representative growth figures for patient 56 for both freshly serial transplanted tumours and for tumours engrafted after storage using both preservation techniques (Fig. 1B,C). After engraftment of the primary tumour in three mice, with a success rate of 5/6 tumour pieces, the second generation was established using the right-sided tumour from mouse 2 (green line). The second generation showed a twofold faster growth rate than the first generation with all mice being sacrificed and all tumours harvested within 100 days after transplantation (Fig. 1B).

### Immunohistochemistry

**Figure 2:**
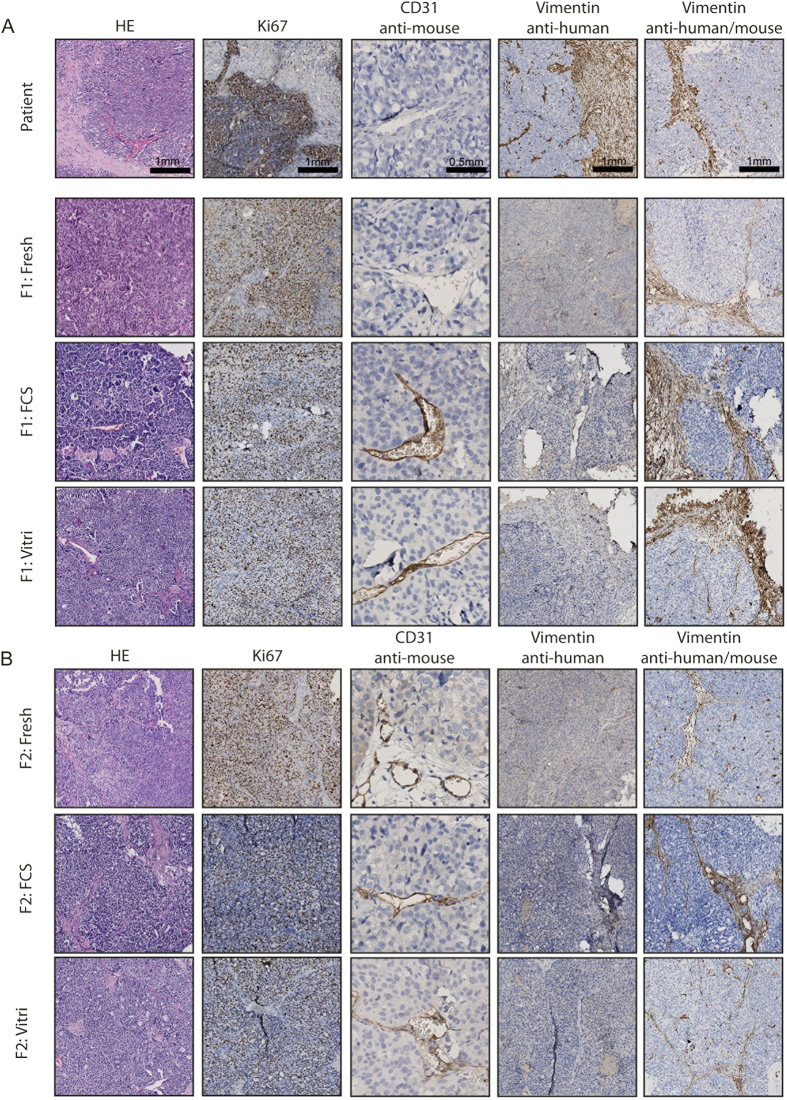
Immunohistochemistry of patient 56 over several generations (F1 **(A)** and F2 **(B)**) and after established growth after storage using either the vitrification (Vitri) or the FCS/DMSO (FCS) protocol on either primary patient tumour tissue (F1) or tumour tissue harvested from previous generations (F2). Magnification 10× and for CD31 20×.

Morphology of the primary patients’ tumour and of tumours engrafted in first and second generations were compared by H&E staining (Fig. 2 and Supplementary Fig. S2–6C). Figure 2, displaying a representative series of H&E stainings for patient 56, shows that through increasing generations there was a tendency towards a more undifferentiated aspect with loss of characteristic histopathological features and increased nuclear atypia (Fig. 2A,B). Stromal infiltration was observed throughout serial transplantation. However, human stroma in the tumours was replaced by mouse stroma. This is shown by loss of human vimentin staining and gain of expression of mouse vimentin, using two vimentin antibodies raised against human and human/mouse, respectively (Fig. 2A,B). Using a monoclonal rat anti-mouse antibody for CD31, we demonstrated increased positive mouse CD31 staining of endothelial cells lining the vessel walls of PDX tumour tissue when compared to the primary tumour, suggesting replacement of human- for mouse vessels. Proliferative rate, as assessed by Ki67, remained high through generations as well as in tissue engrafted after storage for both vitrification and FCS/DMSO (Fig. 2A,B). Expression of Wilm’s Tumour (WT1), known to be primarily expressed in serous ovarian cancers, was seen in all serous patients, and absent in patient 61 (data not shown).

**Figure 3:**
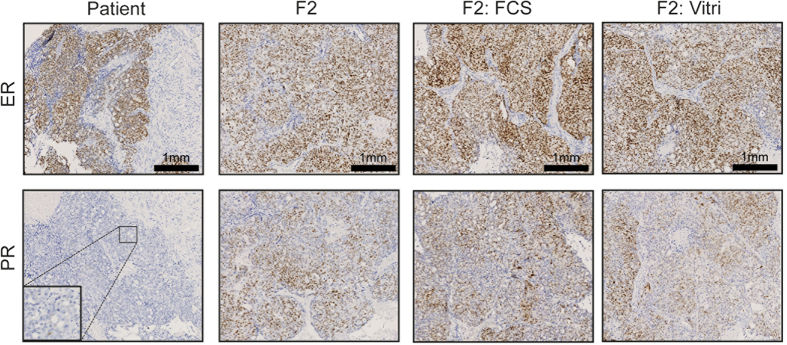
Immunohistochemistry of the ER and PR in representative slides of the primary tumour tissue of patient 56 (Patient), tumour tissue from F2 generation of PDX 56 (F2), and tumour tissue from F2 generation of cryopreserved PDX 56 F1 using either FCS/DMSO (F2:FCS) or vitrification (F2:Vitri). Magnification 10× and 40×.

The oestrogen receptor (ER) and progesterone receptor (PR) are known to be frequently expressed in serous ovarian cancer and are also associated with improved survival^21^. Because of the use of a high percentage of FCS, we hypothesized that FCS containing growth factors may induce a selection on hormone dependent cancer cells and/or changes in signalling pathways. Therefore, we compared the expression of ER and PR between the two storage methods in tumour material from three high-grade serous patients (37, 56 and 67) and one mixed histology ovarian cancer patient (61). Figure 3 shows the expression of ER and PR in tumour tissue from patient 56, tumour tissue from F2 generation of PDX 56, and tumour tissue from F2 generation of cryopreserved PDX 56 generation F1, using either FCS/DMSO or vitrification (Fig. 3). PR expression increased in F2 generations compared with the primary tumour. Neither of the storage methods influenced the expression levels of ER and PR (Fig. 3). In all 4 PDX models, both consistently positive (37, 56 and 67) or negative (61) ER and PR staining was observed through generations.

### Copy number alteration analysis

**Figure 4:**
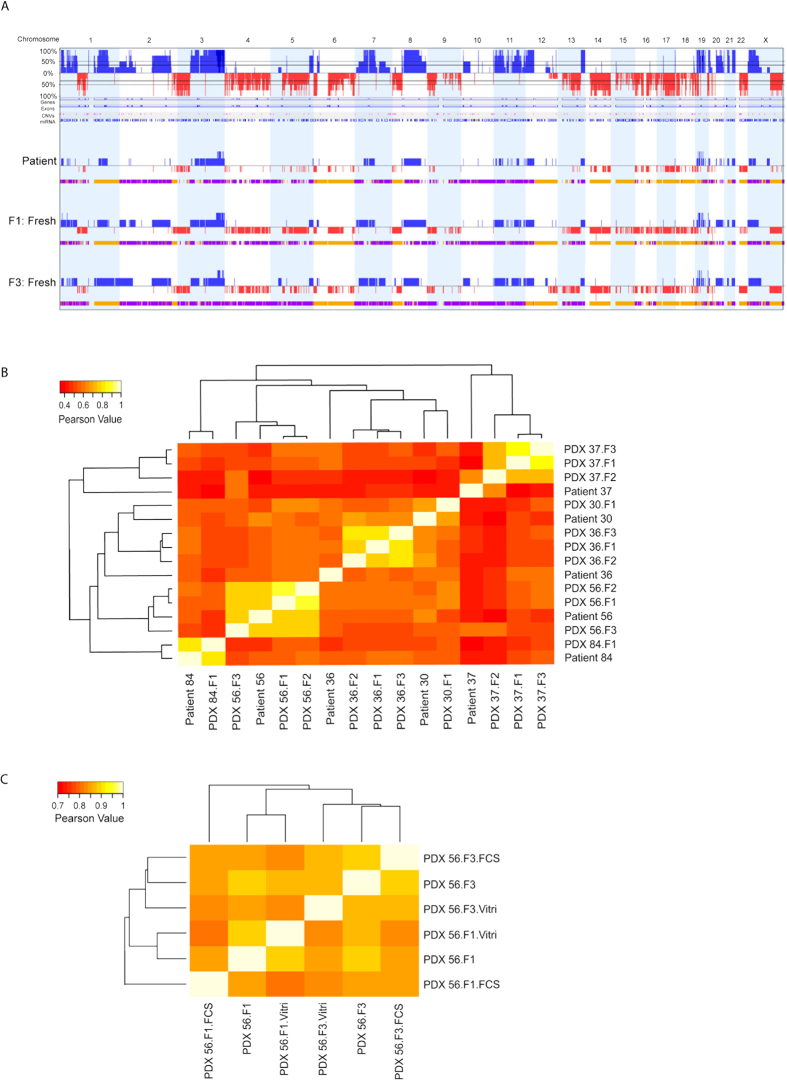
Copy number analysis of ovarian cancer patient tumours and their matched PDX tumours using genome-wide SNP array. (**A**) CNA plots represented the copy number alterations between the primary tumour of patient 56, PDX tumour after first engraftment (F1) and PDX tumour after 3^rd^ engraftment (F3). Genomic gain is indicated in blue and genomic loss is indicated in red over all chromosomes. In the upper CNA plot, the average genomic alteration of all three samples is presented in a similar manner (blue: amplification and red: loss). Below each CNA plot of each sample, the bar with colors represents the allelic events (yellow for loss of heterozygosity (LOH); purple for allelic imbalance). (**B**) Quantitative CNA concordance analysis of tumours of patients and their corresponding PDXs by hierarchical clustering. (**C**) Quantitative CNA concordance analysis of engrafted tumours of patient 56 after preservation using both methods compared to freshly propagated tumours by hierarchical clustering. Note that the scale bar of the Pearson Value is different for (**B** and **C**).

We performed a genome-wide single nucleotide polymorphism (SNP) microarray on tumour material from five independent patients (30, 36, 37, 56 and 84) and their corresponding PDX tumours of different generations (F1, F2 and F3). Besides these samples, bio-banked tumours of PDX 56 using both freezing methods were also included for genotyping analysis. After pre-processing and quality control, resulting data were used to calculate copy number alterations (CNAs) across the entire human genome and were compared among different samples. Four samples from PDX 30 and 84 did not pass quality control and were not included for subsequent analysis. The pattern of CNAs was compared between the primary tumour and different generations of PDX tumours. Grafted tumours maintained the CNA pattern of the parental patient tumour (Fig. 4A and Supplementary Fig. S7). We observed more accumulation of deletion events in the genome of PDX tumours, which seemed to be enhancements of existing genomic aberrations of the primary tumour specimen (Fig. 4A). This could be due to the influence of enrichment of human tumour cells after implantation since mouse stroma replaced the human stroma as aforementioned.

Furthermore, we also determined the concordance of CNAs between tumours from patients and their PDXs (Fig. 4B). A marked heterogeneity was observed among tumours from different patients. However, in general, tumours from the same patient and their established PDXs clustered together. Notably for some PDXs (PDX 36 and 37), genomic consistency was greater among propagated PDX tumours than with the original tumour. This finding again indicates the presence of human stromal and endothelial components in the original tumour from patients that are replaced by murine components during serial propagation in mice. Taken together, the genomic analyses support the notion that ovarian cancer PDX tumours retain their genomic characteristics during propagation over several generations.

Further, no significant copy number changes occurred in the engrafted tumours after storage using both methods, compared to freshly propagated tumours (Fig. 4C and Supplementary Fig. S8A and B). In addition, CNAs were preserved in tumours and showed significantly high concordance, in either stored directly from the patient or from established PDX (Fig. 4C and Supplementary Fig. S8A and B). In conclusion, both biobanking methods, FCS/DMSO and vitrification, did not affect the genomic characteristics of engrafted PDX tumours.

## Discussion

In this study, we presented our extensive panel of 45 ovarian cancer PDXs. Furthermore, we examined two different methods for preserving and thawing of primary ovarian cancer tumour tissue as well as PDX-derived tissue from mice. Overall, we achieved a PDX take rate of 68%. After cryopreservation, we achieved tumour take rates ranging from 38–67% and 67–94% using either a vitrification or FCS/DMSO-based cryopreservation protocol, respectively.

Several studies, focusing on PDX models in different types of cancer, mention cryopreservation or storage of frozen tumour material, either fresh or from propagated xenografts^7^,^22^,^23^,^24^,^25^. However, none of them reported on take- or growth rates or any other outcome after preservation. One of the early studies describing a method for cryopreservation of primary tumour tissue was unsuccessful in all primary cases using colorectal, pancreatic and gastric tumour tissue^26^. Only after cryopreservation of already xenotransplanted tumours, a success rate of 39% was achieved^27^. Although not significantly inferior to storage of tumour pieces of established PDXs, we also observed less efficiency in engraftment of primary frozen patient samples with a longer latency period. Furthermore, this suggests that successful engraftment of frozen tumour tissue is tumour-type dependent.

Sorio *et al*. were the first to describe a successful cryopreservation method for storage of primary pancreatic cancers^28^. A freezing solution consisting of FCS (30%), DMSO (10%) and RPMI (60%) was used and before implantation pieces were soaked in matrigel. Remarkably, take rate in cryopreserved tumours was higher compared to freshly implanted tumours. However, overall take rate per implanted tumour sample was only 21% for cryopreserved tumours, with a reported time of growth of 1–5 months^28^. Further improvement was obtained using FCS (90%) with 10% DMSO for cryopreservation of primary clinical colorectal cancer specimens, resulting in take rates of 71%^29^, in line with our results.

The alternative method, vitrification, is adapted from reproductive medicine, where cryopreservation of ovarian tissue, embryos and oocytes is an important field of study^20^,^30^,^31^. Using vitrification, cells are exposed to different types and concentrations of cryoprotectants in a stepwise manner to avoid extra- and intracellular ice crystals formation-induced damage, followed by a fast direct freezing in liquid nitrogen^30^. Till now, this new vitrification method had not been applied for PDX-derived tumour biobanking or compared with the established FCS-based freezing method. Although being a more time-consuming technique, vitrification of embryos and oocytes has become the method of choice in reproductive medicine with successful conception rates in patients^32^,^33^.

We have further characterized the fresh and biobanked tumours. We showed that over different generations histological and proliferative characteristics grossly remained comparable. Also, no differences were seen between the two freezing protocols. Replacement of original tumour stroma by mouse stroma and takeover of mouse endothelial cells in the vessel lining were seen consistently in all patients after first generation engraftment and subsequent transplantation or storage. The loss of human vimentin and gain of mouse CD31 suggests stromal infiltration and takeover of vascularization by the murine host. This has previously been reported in various types of solid tumours and was shown to occur already in an early phase of engraftment after 4–8 weeks^34^.

Ovarian cancer is known to express both oestrogen and progesterone receptors^21^. Hormone receptor status is dependent on several factors, of which histological subtype is one of them, with the high-grade serous subtype expressing at least one of the two receptors in 84%^21^. Stimulation of especially the ER has shown to enhance ovarian cancer cell proliferation^35^. Furthermore, it has been reported in breast cancer xenografts that ER status is an important factor in tumour take rate^7^,^24^. Therefore, we wanted to examine whether the stimulating growth factors in FCS affected ER and PR expression and thus tumour take rate. However, no differences in immunohistochemical ER and PR staining were observed between tumours derived from fresh and stored tumour tissue, suggesting that neither storage nor the percentage of FCS in the freezing solution influenced the hormone receptor status of these ovarian cancer PDXs.

It has been well established that there is a high concordance between primary tumour and tumours taken from various generation of PDXs in term of genomic alterations^36^,^37^. However, an accumulation of genomic alterations in the PDX compared with the patient tumours was also described^7^,^38^. The main contributor for these genomic alterations could be the enrichment of human tumour DNA after loss of human stromal cells during propagation in mice. A recent whole-genome study of breast cancer patient tumours, PDX tumours and their lymphocytes, using exome sequencing and RNA-sequencing analysis, showed genomic stability of PDX tumours during serial transplantation in mice^39^. In alignment with previous results, we found the same trend of enhancement of certain genomic aberrations that were predisposed in patient tumour with a lower frequency. Further, the prominent CNA patterns were typically maintained in the engrafted tumours after storage using both protocols when compared to freshly propagated tumours. These genotyping results support our immunohistochemically confirmed phenotypic data of PDXs tumours, which showed no differences in histological and proliferative characteristics between two freezing protocols.

Ovarian cancer PDX models are nowadays well established and examined for their patients’ mimicking potential^10^,^40^,^41^. The next step will be to use these models for development of patient tailored therapy, either in pre-clinical drug testing with new targeted drugs or as a model for patients’ personalized therapy decision making^36^. For logistic purposes and feasibility of these trials, biobanking of tumour tissue will be essential. Currently, we implant fresh tumour material in F1, after which harvested tumours are stored using the FCS/DMSO method. By doing this, we reduce loss of precious patient tumor material and maximize the chance of successful establishment of primary patient material as a PDX. Furthermore, since these tumors show high take rates in F2 and relatively fast growth along with recapitulating most of the histological and genomic features of patient tumours, we are able to largely expand our biobank with high quality reimplantable tumour material. With this protocol as a standard of biobanking, we have achieved reproducible take rates for experimental purposes.

In conclusion, we established an extensive panel of 45 ovarian cancer PDXs, reflecting all major histological subtypes. We show that two protocols containing either high or low FCS can be used for biobanking of ovarian cancer and PDX tissues. However, the FCS/DMSO-based cryopreservation protocol has been proven to be more successful with higher tumour tissue take rates. Primary engraftment of fresh patient-derived tumours in mice followed by freezing of successfully established PDXs is the preferred way of ovarian cancer PDX biobanking.

## Methods

### Patients and tumour samples

Ovarian cancer specimens were obtained during surgery, before or after 3 cycles of a carboplatin-taxol chemotherapy regime. Before surgery, all patients gave written informed consent for their tumour samples to be used for research. Clinicopathological data, obtained during standard treatment and follow-up, were stored in an anonymous database managed by two dedicated data managers. This study was approved by the medical ethics committee of the University Medical Centre Groningen and carried out in accordance with the approved guidelines and regulations.

### Establishing of tumour xenografts

Specimens obtained during surgery were transported in transportation media consisting of DMEM containing 10% FCS, 1% penicillin/streptavidin, 2.5 μg/mL Fungizone and 50 μg/mL Gentamycin on room temperature. Within 5 hours, tumour fragments were cut into pieces of ca. 3 × 3 × 3mm using sterile surgical instruments. One piece was snap-frozen in liquid nitrogen and another piece was formalin-fixed for later histological examination. Typically, 2 pieces were subcutaneously implanted on both sides of the flank of three 6–12 weeks old female NOD.Cg-Prkdcscid Il2rgtm1Wjl/SzJ mice (internal breed, Central Animal Facility, University Medical Centre Groningen). Surgery was performed under sterile conditions in a laminar flow cabinet using sterilized surgical instruments. A single cut was made in the neck of the animal and two pieces were subcutaneously transferred to either side of the flank using blunt forceps. Remaining pieces were preserved using vitrification and/or FCS/DMSO protocol. Mice were kept under pathogen-free conditions in the Central Animal Facility (University Medical Centre Groningen) and received sterilized food and water *ad libitum*. All animal experiments were approved by the Institutional Animal Care and Use Committee of the University of Groningen (Groningen, the Netherlands) and carried out in accordance with the approved guideline “code of practice: animal experiments in cancer research”.

### Tumour growth

Upon growth, tumours were measured once or twice a week in two dimensions using a slide vernier calliper. Tumour volume was calculated using the equation (width^2^xlength)/2. When tumour size reached >1 cm^3^ or animals reached one of the other endpoints as mentioned in the Dutch Code of Practice for animals experiments in cancer research (Netherlands Inspectorate for Health Protection, Commodities and Veterinary Public Health, 1999), tumours were harvested and put in transportation media, for either direct propagation into a further generation or for storage. Latency time, the time till growth was observed, was defined as the time between implantation and the first moment of measurable tumour (approximately 70 mm^3^).

### Preservation using vitrification and thawing procedure

Primary tumours and tumours harvested from established PDXs were cut into pieces of ca. 3 × 3 × 3mm using sterile surgical instruments in a laminar flow cabinet. Sterile 24-well plates were prepared either containing 1 mL of rinse medium or 1 mL of either vitrification solution 1 or 2 (VS1 or VS2). Another 24-well plate, containing VS3, was prepared on ice. Constitution of different solutions is shown in Supplementary Table S3. All solutions were filtered through a sterile 0.2 μm filter. Each tissue fragment was first incubated in rinse medium at room temperature for 5 minutes. Subsequently, pieces were transferred to VS1 and then to VS2 with an incubation time of 5 and 10 minutes at room temperature, respectively. Afterwards, pieces were transferred into VS3 and incubated on ice for 10 minutes. Finally, each piece was transferred into a sterile cryotube, snap-frozen in liquid nitrogen and then stored in a liquid nitrogen tank.

For thawing, cryotubes were held in a water bath (37 °C) until melted. In a laminar flow cabinet, sterile 24-well plates were prepared with wells containing either 1 mL of thawing solution (TS) 1, 2, 3 or 4 (Supplementary Table S3). All solutions were filtered through a sterile 0.2 μm filter. Tissue fragments were placed in TS1 for 2 minutes at room temperature. Then pieces were transferred for 5 minutes in TS2, TS3 and TS4, respectively. Finally, pieces were kept in transportation media at room temperature until implantation.

### Preservation using FCS/DMSO and thawing procedure

After harvesting and dissecting, primary tumours and tumours harvested from established PDXs were cut into pieces of ca. 3 × 3 × 3 mm^3^ using sterile surgical instruments in a laminar flow cabinet. Pieces were transferred into sterile cryotubes containing 1.5 mL 95%FCS/5% DMSO. Cryotubes were put in a freezing container containing isopropanol, placed in an −80 °C freezer overnight and transferred to liquid nitrogen storage the next day.

For thawing, cryotubes were held in a water bath (37 °C) until melted. In a laminar flow cabinet pieces were dipped into FCS for 2 minutes and were then transferred into transportation media at room temperature until implantation.

### Immunohistochemistry

For immunohistochemistry, 4 μm sections were cut from paraffin-embedded tumour tissue and these sections were mounted on amino-propyl-ethoxy-silan-coated glass slides. Morphology of tumours was assessed using staining with haematoxylin and eosin (H&E) and immunohistochemical staining for Ki67, CD31, WT1, Vimentin (anti human clone, anti human/mouse), ER and PR. Antigen retrieval methods, primary antibodies and detection methods are presented in Supplementary Table S4. Colon, kidney and tonsil served as positive controls for vimentin, WT-1 and CD31, respectively. Oestrogen- and progesterone- receptor positive breast cancer served as positive controls for ER and PR staining. All slides were deparaffinised in xylene. Endogenous peroxidase was blocked by incubation with 0.3% hydrogen peroxidase for 30 minutes. Lastly, staining was visualized by DAB and counterstaining was performed with haematoxylin. ER and PR status was considered positive if at least 1% of tumour nuclei stained positive, according the official cut-off determined by the American Association of Clinical Oncology (ASCO)^42^. To obtain more information about the percentage of tumour cells in tumour pieces implanted, formalin-fixed tumour pieces obtained at the time of surgery of patients, were stained for H&E and used as a representative for implanted pieces. Percentage of tumour cells was scored using a 10× magnification, scoring 3 fields of interest, by two independent observers. A mean percentage of tumour cells was calculated and differences between established and non-established PDXs was calculated using an unpaired two-tailed Student’s t-test.

### DNA Isolation of Patient and PDX tumours

Representative frozen blocks of each patient tumour and their corresponding PDX tumours of different generation, were retrieved for DNA extraction. Histological slides from the frozen tissue were taken for quantifying amount of vital tumour cells. Frozen sections of 10 um were cut with periodic 4 μm sections for H&E staining. For some samples, slides were macro-dissected to obtain >85% neoplastic cells. DNA of all samples was isolated using standard salt-chloroform extraction and isopropanol precipitation. In the end, precipitated DNA was re-suspended in Tris-EDTA buffer (10 mM Tris; 1 mM EDTA, pH 8.0). Genomic DNA was amplified in a multiplex PCR according to the BIOMED-2 protocol, to check the DNA’s structural integrity^43^.

### SNP array-based genotyping

Genome-wide single nucleotide polymorphism (SNP) genotyping for 5 independent patients (30, 36, 37 and 56 and 84) along with their corresponding PDX tumours was performed on Illumina HumanOmniExpressExome8^R^ BeadChip containing over 900,000 markers. Genotypes were called with the standard algorithm provided by Illumina and implemented in Genome Studio software with Genotyping module. All the samples were passed the inclusion quality control (QC) criteria including the limit of not >5% of missing genotyping. Additionally, SNPs of samples derived from the patient and the corresponding PDX tumours were also compared with SNPs of normal leucocytes of a healthy blood donor. In addition, NSG mouse tail and liver DNA was included on the same SNP array as technical controls to check the specificity and cross-reactivity of the array. Further advanced analysis was performed with Nexus Copy number^R^ software (BioDiscovery) using standard SNPs frequency significance testing and enrichment analysis to generate CNA profiles. Moreover, quantitative CNA correlative analysis of patients and corresponding PDXs were performed as described previously^44^. Briefly, processed array data were binned into numerical integer value ranging from 1 to 5 for each of the quality controlled passed SNP probes (n = 906411), where 1 and 2 indicated copy number losses, whereas 4 and 5 were defined as calls for copy number gains. Subsequently, a copy number call matrix was formed and hierarchical clustering was performed with the use of Pearson correlation metrics and average linkage to reveal similar clusters. All the computations and heatmap generation were performed in the R Statistical Environment (R version 3.1.1, R Development Core Team, foundation for Statistical Computing, Vienna, Austria).

### Statistics

All statistical analyses were performed using GraphPad version 5.01 (GraphPad Software, http://www.graphpad.com). Significance between take rates was compared with the Fisher’s exact test. Differences between established and non-established PDXs was calculated using an unpaired two-tailed student’s t-test. For all tests, *P* values < 0.05 were considered statistically significant.

## Additional Information

**How to cite this article**: Alkema, N. *et al*. Biobanking of patient and patient-derived xenograft ovarian tumour tissue: efficient preservation with low and high fetal calf serum based methods. *Sci. Rep*. **5**, 14495; doi: 10.1038/srep14495 (2015).

## Supplementary Material

Supplementary Information

## Figures and Tables

**Table 1:** Successfully established primary ovarian cancer PDXs from May 2011 till January 2015.

Histology	Nr of established PDXs	PDX success rate	Latency time in days Median (range)
Serous	31	31/43 (72%)	82 (14–270)
Endometrioid	7	7/10 (70%)	56 (10–105)
Clear cell	3	3/5 (60%)	21 (16–30)
Mucinous	1	1/2 (50%)	17 (15–21)
Mixed phenotype	3	3/6 (50%)	40 (30–60)
**Total**	45	45/66 (68%)	43 (10–270)

**Table 2:** Take rate of fresh implanted primary tumour pieces and implanted tumour pieces after preservation via vitrification and/or FCS/DMSO for all different PDXs.

PDX number (Histology)		F1	Mean latency time in days	F2	Mean latency time in days
30 (Serous)	Direct propagation	4/6	150 (90–270)	2/2	75 (46–104)
	Vitrification	2/6	170 (154–186)	—	—
36 (Serous)	Direct propagation	1/6	115	6/6	30 (19–39)
	Vitrification	—	—	3/6	55 (35–58)
	FCS/DMSO	—	—	2/2	20
37 (Serous)	Direct propagation	4/6	71 (50–90)	6/6	88 (75–100)
	Vitrification	4/6	257 (170–320)	7/9	115 (97–155)
	FCS/DMSO	—	—	6/7	60 (35–78)
56 (Serous)	Direct propagation	4/6	71 (40–192)	3/4	60 (55–70)
	Vitrification	1/3	220	2/3	90 (80–100)
	FCS/DMSO	3/3	140 (80–220)	6/7	40 (25–70)
61 (Mixed)	Direct propagation	6/6	40 (30–44)	4/4	15 (7–30)
	Vitrification	1/3	70	1/3	60
	FCS/DMSO	3/3	65 (18–160)	3/3	15 (10–20)
67 (Serous)	Direct propagation	2/6	210 (170–250)	4/4	55 (40–86)
	Vitrification	0/3	—	3/3	95 (75–140)
	FCS/DMSO	0/3	—	3/3	90 (75–115)
84 (Serous)	Direct propagation	2/4	58 (55–62)	2/4	25 (20–30)
	FCS/DMSO	—	—	6/6	42 (20–59)
157 (Endometrioid)	Direct propagation	4/4	12 (10–14)	4/4	10 (8–15)
	FCS/DMSO	—	—	6/6	25 (14–44)

Abbreviations: FCS = Fetal Calf Serum, DMSO = Dimethyl sulfoxide, F = generation number, PDX = patient-derived xenograft.
