# Supplementary material for: Biobanking of patient and patient-derived xenograft ovarian tumour tissue: efficient preservation with low and high fetal calf serum based methods
Source: Sci Rep. 2015 Oct 6;5:14495. doi: 10.1038/srep14495 (PMC4594124; doi:10.1038/srep14495)
Supplement: Supplementary Information [file srep14495-s1.pdf]

Supplementary information

Biobanking of patient and patient-derived xenograft ovarian tumour tissue: efficient preservation with low and high fetal calf serum based methods.

Nicolette Alkema, Tushar Tomar, Evelien Duiker, Gert Jan Meersma, Harry Klip, Ate van der Zee, Bea Wisman, Steven de Jong

Supplementary figure 1

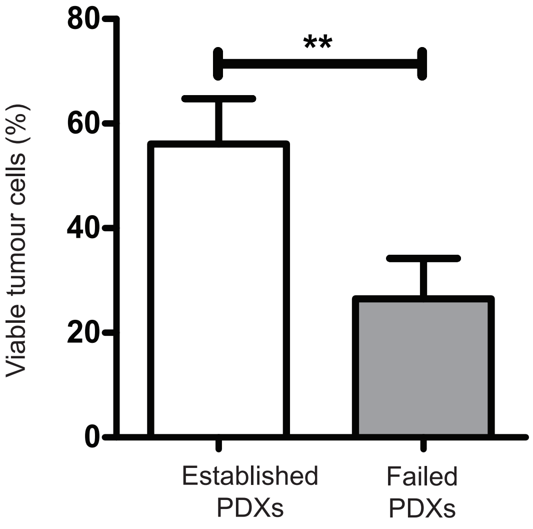

Supplementary figure S1. Percentage of tumour cells in H&E's obtained from representative tumour pieces of PDXs that either successfully engrafted or failed to engraft. **= $p < 0.01$.

Supplementary figure 2

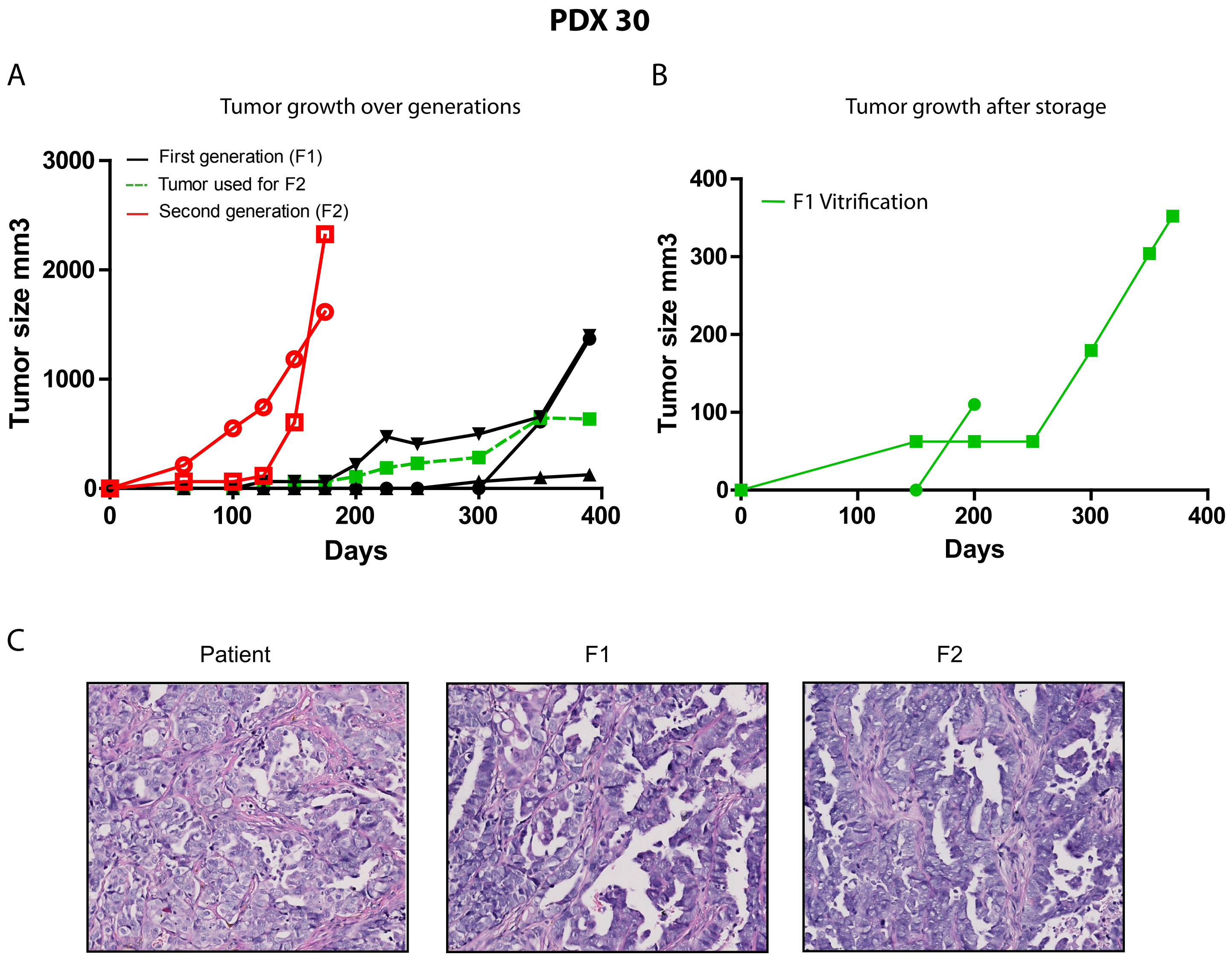

Supplementary figure S2. (A) Tumour growth of fresh implanted tumour tissue from patient 30 and further propagation of the tumour (green line) into the second generation (red lines). **(B)** Tumour growth of stored and subsequently thawed and re-implanted tumour tissue from patient 30. Tumour tissue was directly frozen after patients' primary surgery (F1) using the vitrification protocol. **(C)** Representative H&E staining of patient 30 over several generations (F1 and F2). Magnification 10x.

Supplementary figure 3

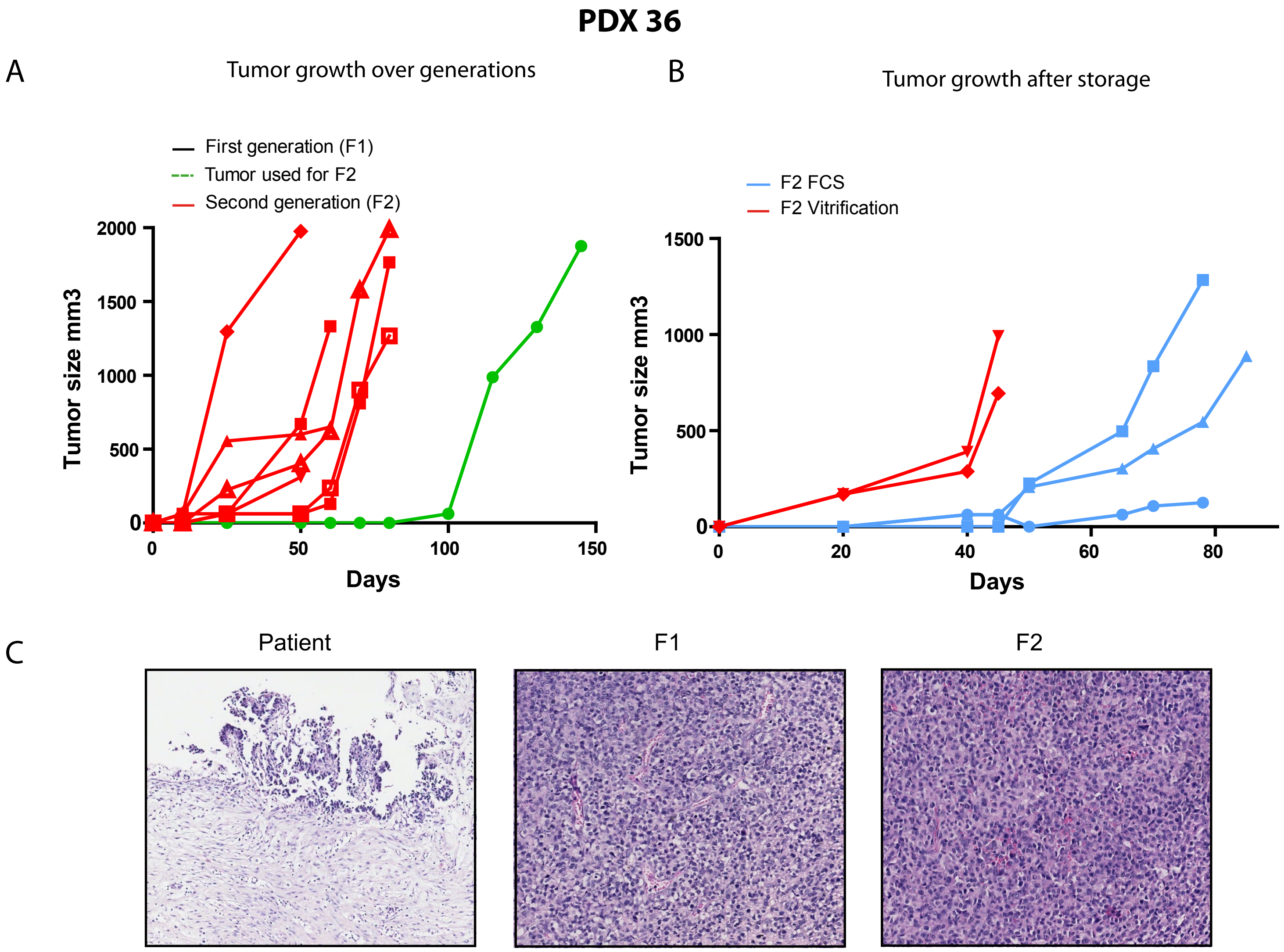

Supplementary figure S3. (A) Tumour growth of fresh implanted tumour tissue from patient 36 and further propagation of the tumour (green line) into the second generation (red lines). **(B)** Tumour growth of stored and subsequently thawed and re-implanted tumour tissue from patient 36. After establishment of a PDX, tumour tissue was harvested from the mouse (F2) and frozen using either the vitrification (red line) or FCS/DMSO (blue line) protocol. **(C)** Representative H&E staining of patient 36 over several generations (F1 and F2). Magnification 10x.

Supplementary figure 4

PDX 37

A

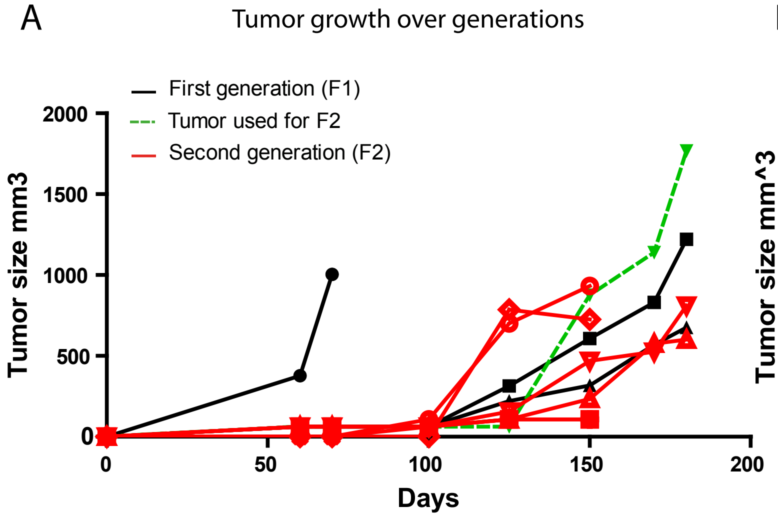

B

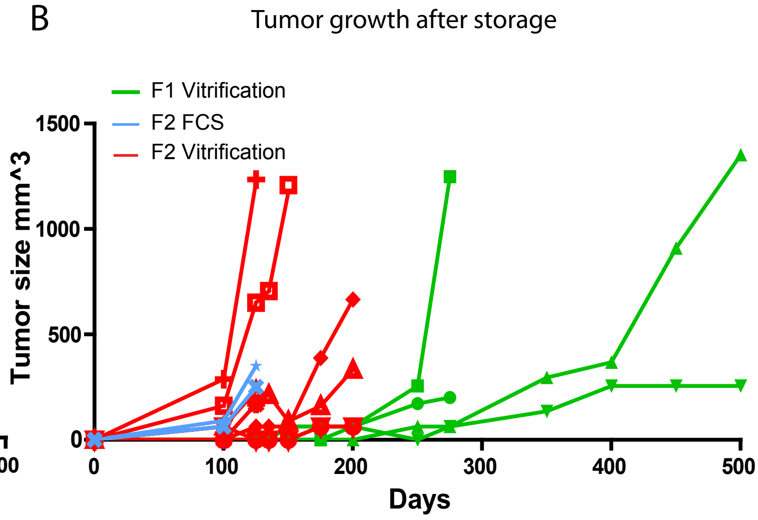

C

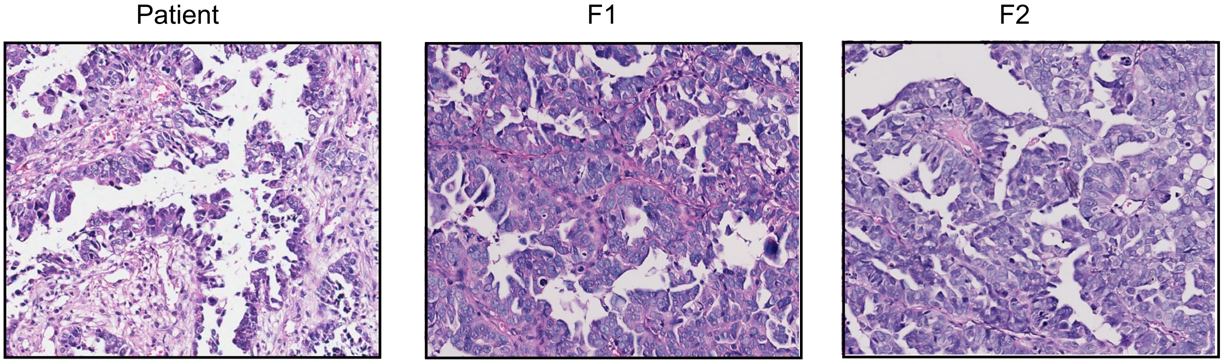

Supplementary figure S4. (A) Tumour growth of fresh implanted tumour tissue from patient 37 and further propagation of the tumour (green line) into the second generation (red lines). **(B)** Tumour growth of stored and subsequently thawed and re-implanted tumour tissue from patient 37. Tumour tissue was either directly frozen after patients primary surgery (F1) using the vitrification (green line) protocol. After establishment of a PDX, tumour tissue was harvested from the mouse (F2) and frozen using either the vitrification (red line) or FCS/DMSO (blue line) protocol. **(C)** Representative H&E staining of patient 37 over several generations (F1 and F2). Magnification 10x and 40x.

Supplementary figure 5

PDX 61

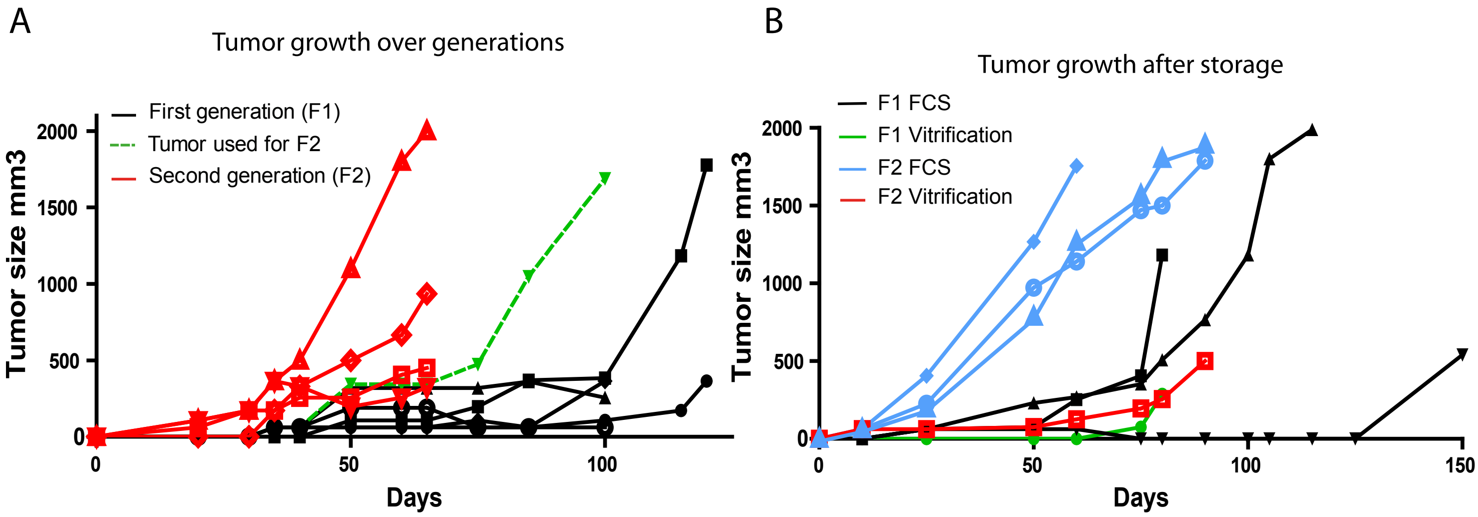

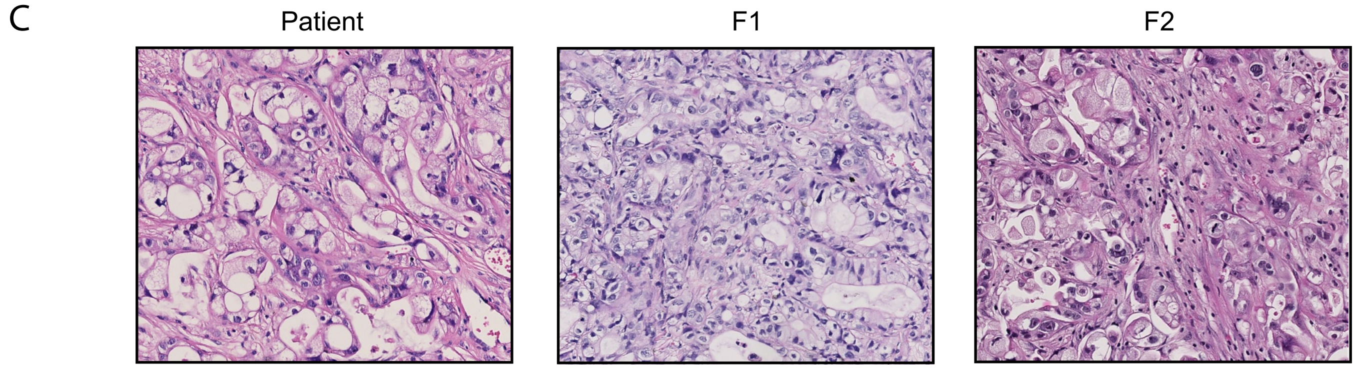

Supplementary figure S5. (A) Tumour growth of fresh implanted tumour tissue from patient 61 and further propagation of the tumour (green line) into the second generation (red lines). **(B)** Tumour growth of stored and subsequently thawed and re-implanted tumour tissue from patient 61. Tumour tissue was either directly frozen after patients primary surgery (F1) using either the vitrification (green line) or FCS/DMSO (black line) protocol. After establishment of a PDX, tumour tissue was harvested from the mouse (F2) and frozen using either the vitrification (red line) or FCS/DMSO (blue line) protocol. **(C)** Representative H&E staining of patient 61 over several generations (F1 and F2). Magnification 10x.

Supplementary figure 6

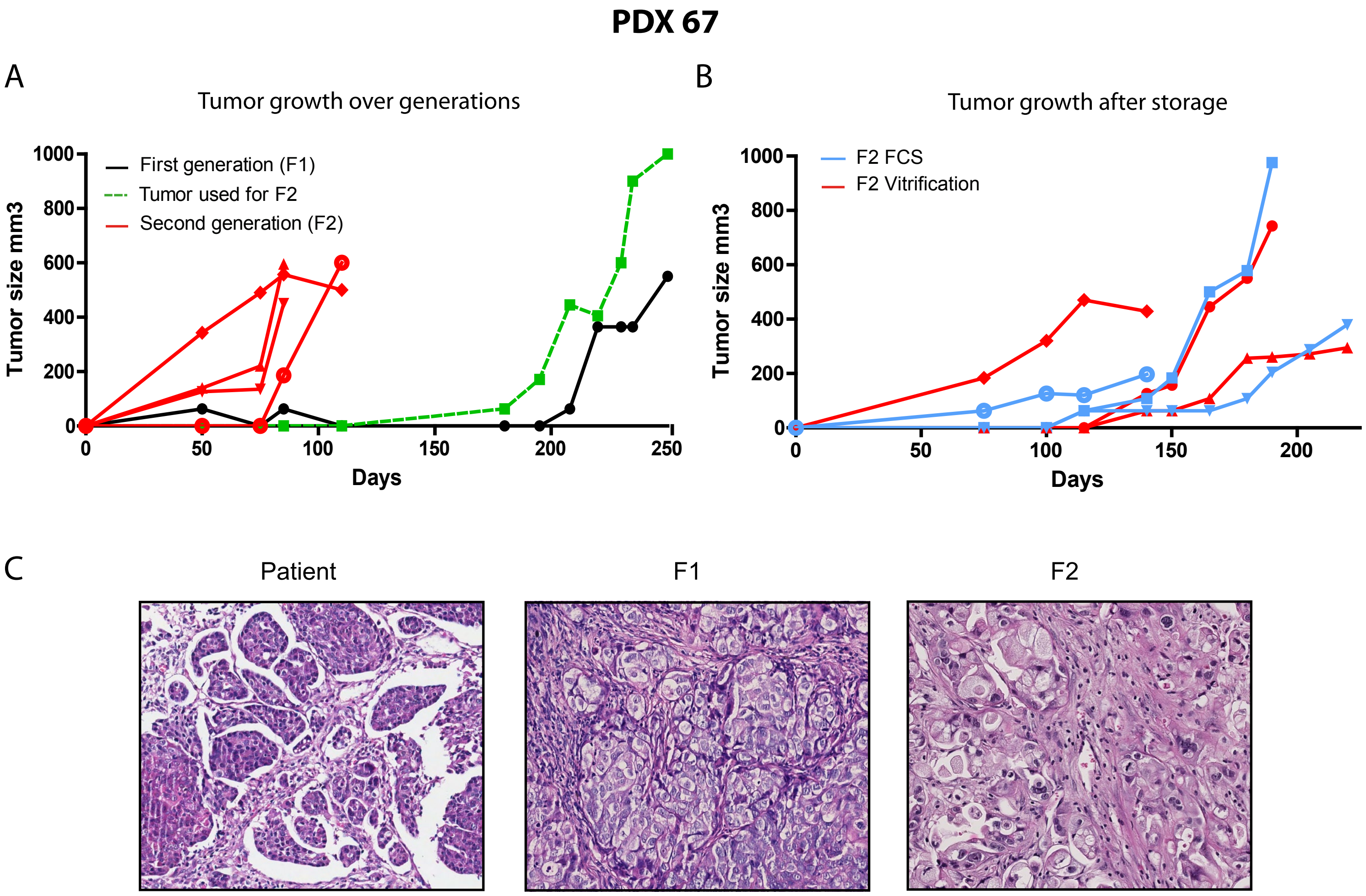

Supplementary figure S6. (A) Tumour growth of fresh implanted tumour tissue from patient 67 and further propagation of the tumour (green line) into the second generation (red lines). **(B)** Tumour growth of stored and subsequently thawed and re-implanted tumour tissue from patient 67. After establishment of a PDX, tumour tissue was harvested from the mouse (F2) and frozen using either the vitrification (red line) or FCS/DMSO (blue line) protocol. **(C)** Representative H&E staining of patient 67 over several generations (F1 and F2). Magnification 10x.

Supplementary figure 7

A

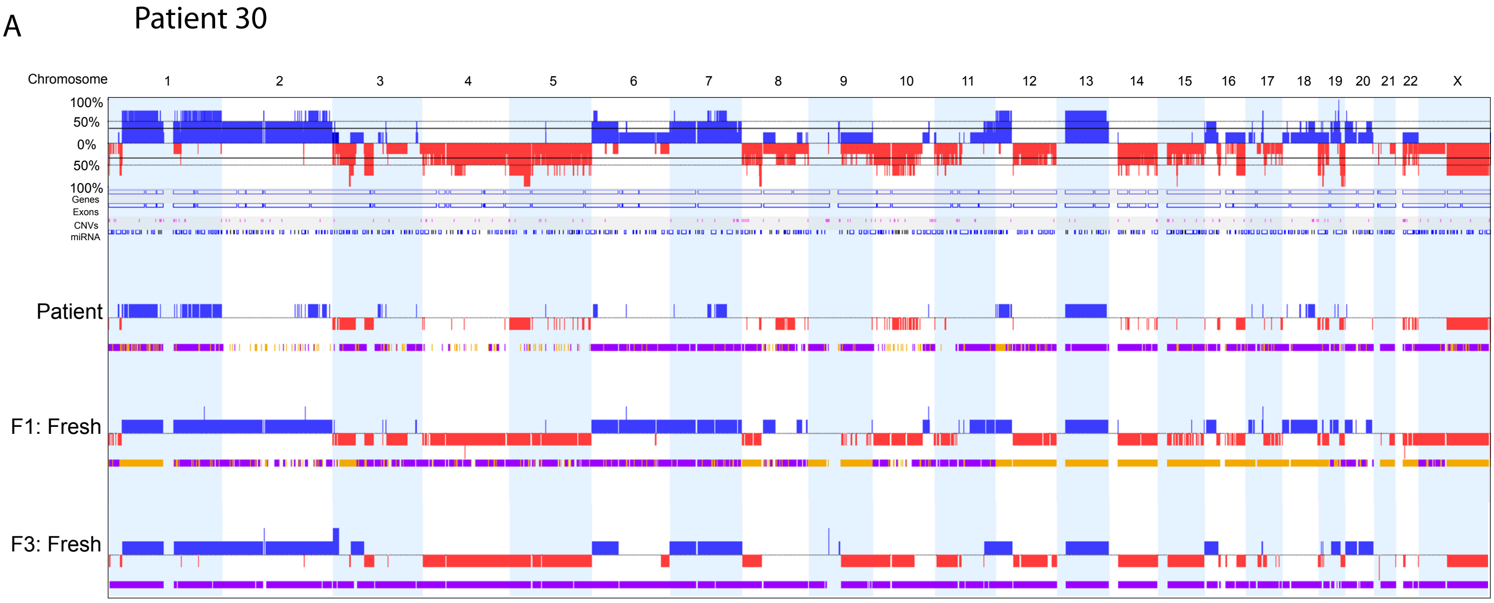

B

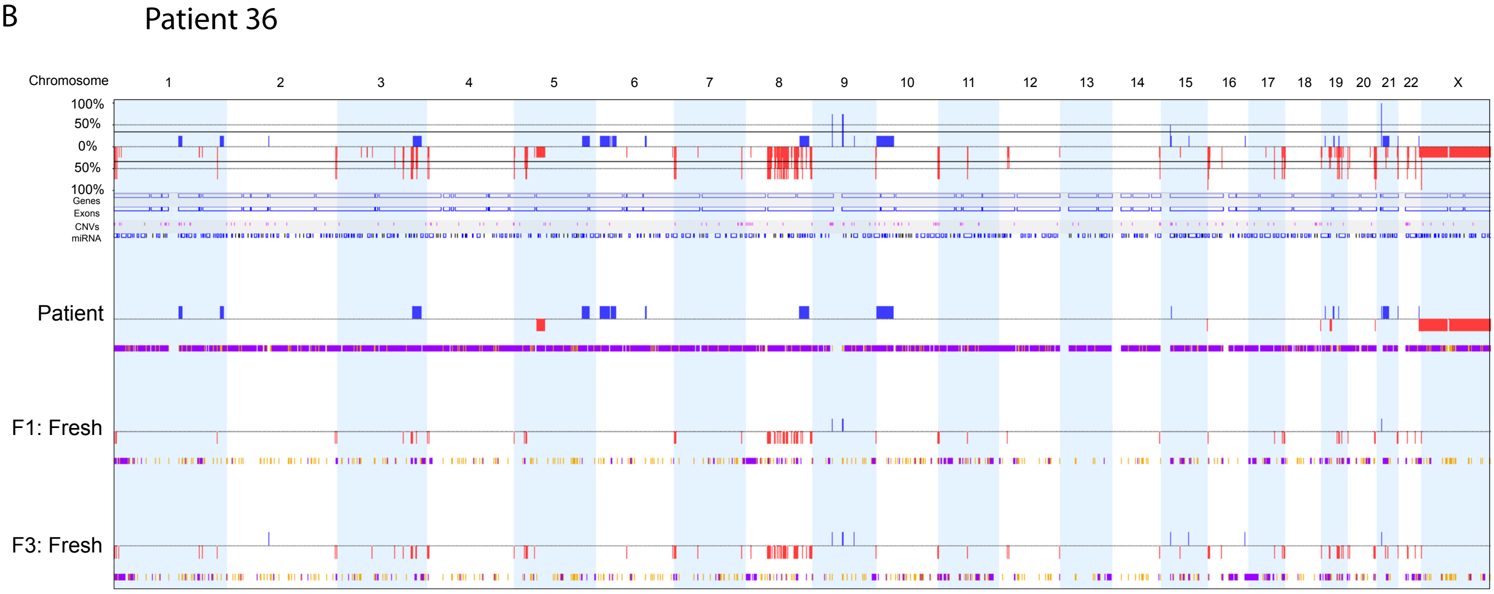

C

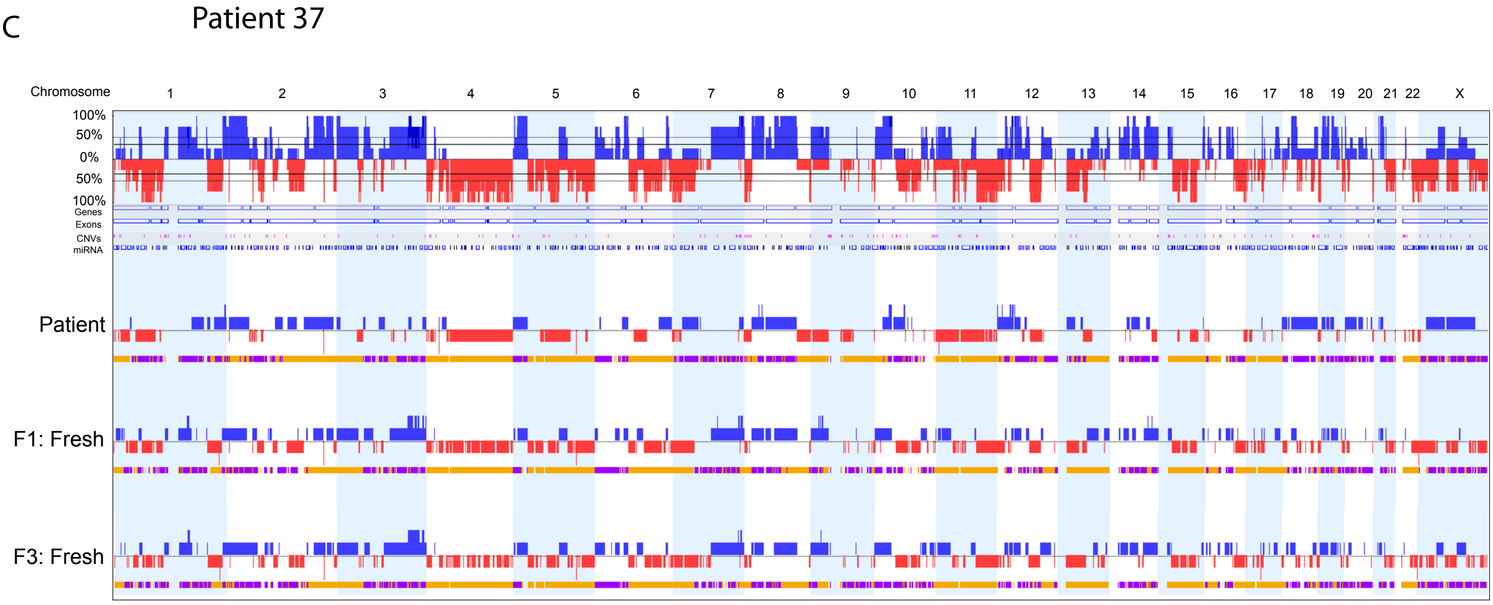

Supplementary figure S7. Copy number alterations (CNAs) analysis of PDX tumors of patient 30 (Suppl. Fig. 6A), 36 (Suppl. Fig. 6B) and 37 (Suppl. Fig. 6C) using genome-wide SNP array. CNA plots represented the copy number alterations between the primary tumour of the patient, PDX tumour after first engraftment (F1) and PDX tumour after third engraftment (F3). Genomic gain is indicated in blue and genomic loss is indicated in red over all chromosomes. At top of each comparative CNA plot for each sample, the average genomic alteration of all three samples is presented in similar manner (blue: amplification and red: loss). Below each CNA plot of each sample, the bar with colours represents the allelic events (yellow for loss of heterozygosity (LOH); purple for allelic imbalance).

Supplementary figure 8

A

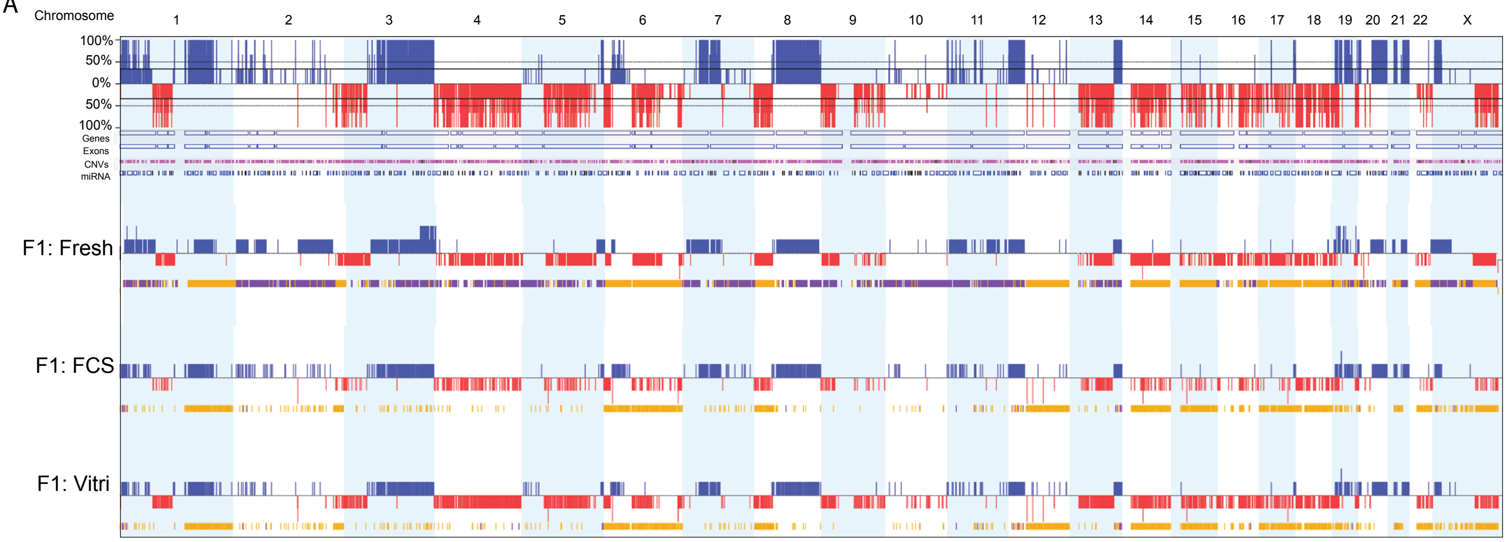

B

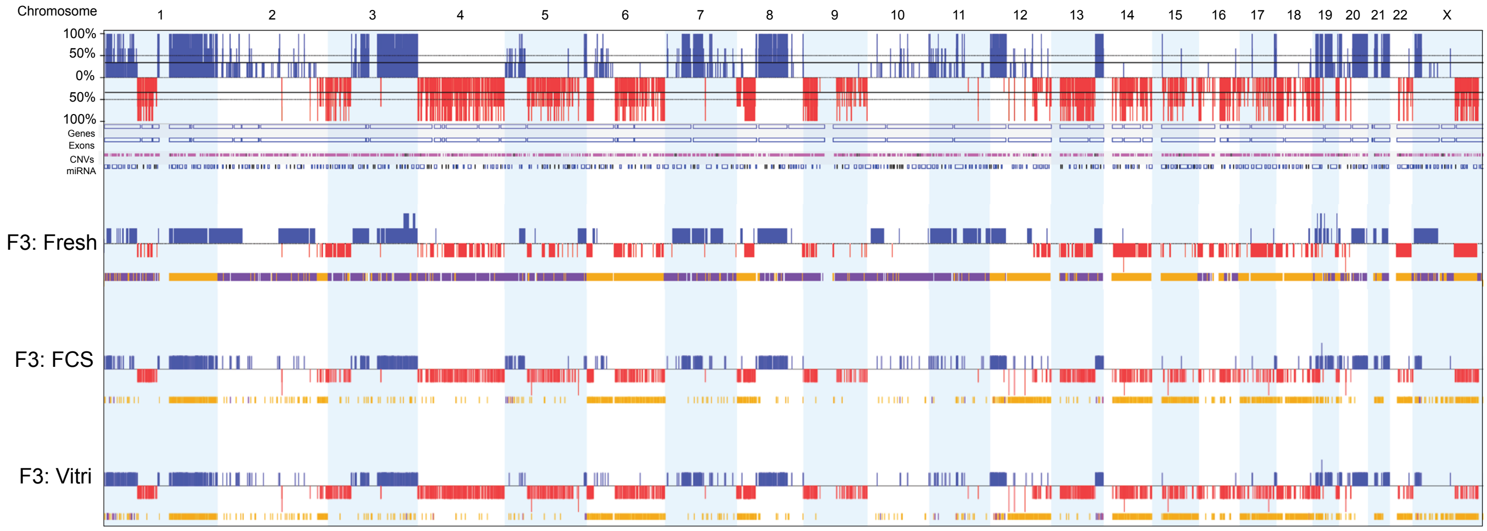

Supplementary figure S8. Copy number analysis (CNA) of PDX tumors of patient 56 using genome-wide SNP array. Comparative CNA plots of biobanked PDX tumor by FCS/DMSO and vitrification methods to their corresponding freshly implanted tumors either from human patients directly (F1) **(A)**, or from serial transplantation from mouse (F3) **(B)**. Genomic gain is indicated in blue and genomic loss is indicated in red over all chromosomes. At top of each comparative CNA plot for each sample, the average genomic alteration of all three samples is presented in similar manner (blue: amplification and red: loss). Below each CNA plot of each sample, the bar with colors represents the allelic events (yellow for loss of heterozygosity (LOH); purple for allelic imbalance).

Supplementary figure 9

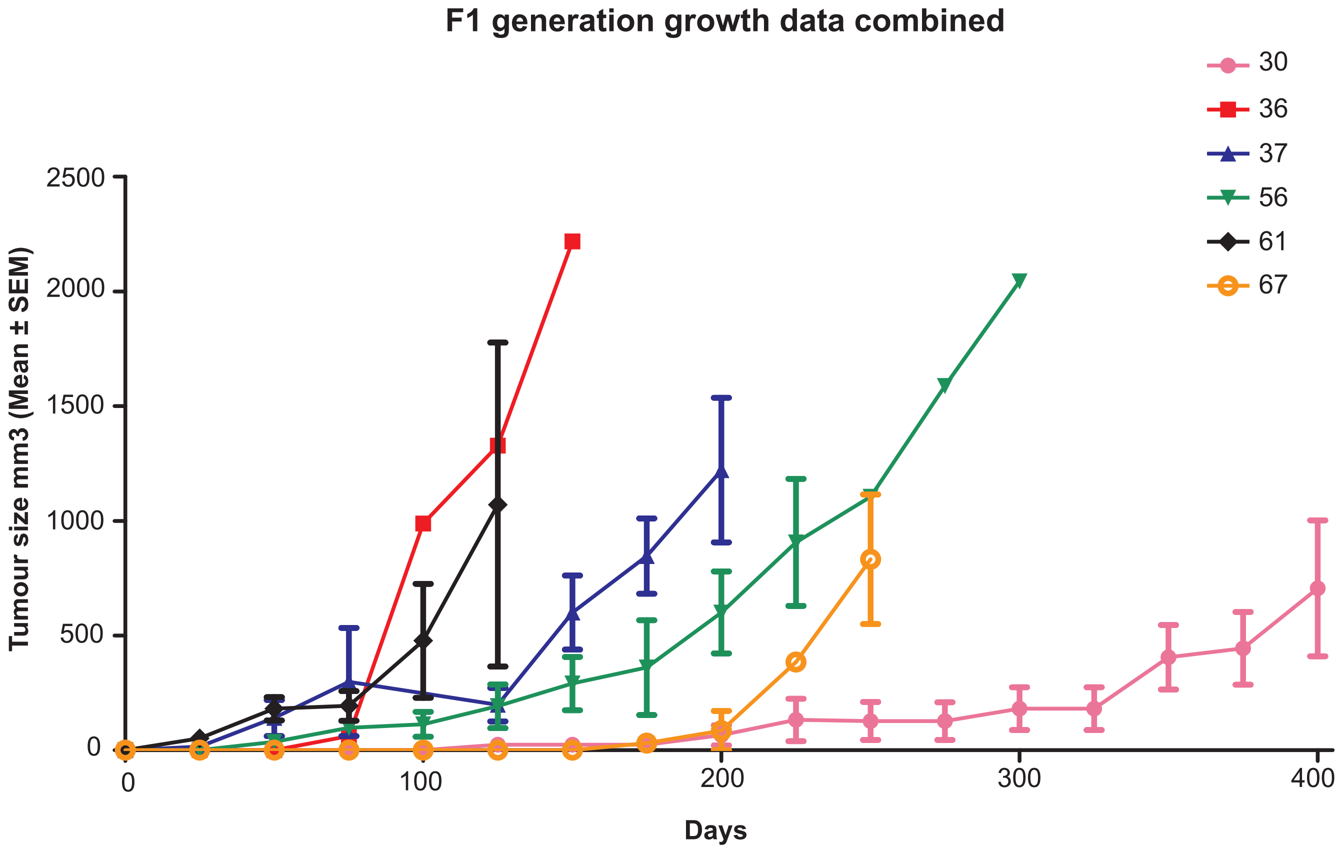

Supplementary figure S9. Growth curves of 6 of the PDXs in F1 generation, depicted as mean of all growing tumours combined ± SEM.

Supplementary tables

Supplementary table 1. Complete list of established ovarian cancer PDXs including histological subtype, time of tissue collection and status at last follow-up.

PDX	Histological subtype	Grade	Stage	Tissue obtained at primary or interval debulking or at relapse	Status at last follow-up
30	Serous	High	IIIC	Primary	Relapse after 24 months
36	Serous	High	IIIC	Primary	Relapse after 18 months
37	Serous	High	IIIC	Primary	Disease-free at last FU
56	Serous	High	IIIC	Interval	Disease-free at last FU
60	Mucinous	High	IIA	Primary	No response, deceased
61	Mixed	NA	IV	Primary	No response, deceased
67	Serous	High	IIIC	Primary	Disease-free at last FU
68a	Serous	High	IIC	Primary	Relapse after 26 months
68b	Serous	High	NA	Relapse 68a	Re-debulking
70	Serous	High	IIIA	Primary	Disease-free at last FU
79	Serous	Low	IIIC	Primary	No response, deceased
81	Endometrioid	Moderate	IC	Primary	Disease-free at last FU
84	Serous	High	IV	Primary	Disease-free at last FU
112	Endometroid	High	IIIC	Relapse	Disease-free at last FU
130	Serous	High	IIIB	Primary	Disease-free at last FU
137	Serous	High	IV	Interval	Disease-free at last FU
143	Serous	High	IIIC	Interval	No FU
155	Clear cell	NA	IIIC	Primary	No response, deceased
157	Endometrioid	High	IV	Primary	No response, deceased
158	Serous	High	IC	Interval	No FU
160	Serous	High	IV	Primary	Palliative treatment
167	Endometrioid	Low	IV	Interval	Moderate response
169	Serous	High	IIIC	Interval	Relapse after 14 months
171	Serous	High	III	Relapse	Relapse after 37 months
174	Serous	High	IV	Interval	Disease-free at last FU
176	Serous	High	IIIC	Primary	Disease-free at last FU
177	Serous	High	IIC	Primary	Disease-free at last FU
179	Serous	High	IIIC	Interval	No response, deceased
180	Clear cell	NA	IIIC	Interval	No response, deceased
183a	Serous	High	IIIC	Primary	Disease-free at last FU
183b	Serous	High	NA	Relapse 183a	Relapse after 11 months
187	Serous	High	IIIC	Interval	Good response
188	Serous	High	IIIC	Primary	No response, deceased
189	Endometrioid	High	IC	Primary	Disease-free at last FU
191	Mixed	NA	IIIC	Primary	Disease-free at last FU
193	Serous	High	IIIC	Primary	Disease-free at last FU
195	Serous	High	IV	Primary	Disease-free at last FU
200	Serous	High	IIIC	Interval	No response, deceased
202	Serous	Moderate	IIIC	Interval	Good response
203	Serous	High	IIIC	Primary	Disease-free at last FU
207	Serous	High	IIIC	Primary	Disease-free at last FU
208	Endometrioid	Low	IIB	Primary	Disease-free at last FU
225	Mixed	NA	IIIC	Primary	Disease-free at last FU
229	Endometrioid	Low	IC	Relapse	Relapse after 30 months
237	Clear cell	NA	IIIC	Primary	Progressive disease

All patients received either neo-adjuvant or adjuvant carboplatin/taxol chemotherapy. Abbreviations: NA=non-applicable, FU=follow-up.

Supplementary table 2. Take rate of fresh implanted primary tumour and implanted tumour after preservation via vitrification and/or FCS/DMSO in 8 selected PDXs.

Generation	F1	Latency time spread	F2	Latency time spread
**Take rate tumour pieces (n= tumour pieces):**				
**Fresh propagation**	27/44 (61%)	10-270 days	31/34 (91%)	7-104 days
**Vitrification**	8/21 (38%)	70- 320 days	16/24 (67%)	35-155 days
**FCS/DMSO**	6/9 (67%)	18-220 days	32/34 (94%)	10-115 days
**Established PDX (n= patients):**				
**Fresh propagation**	8/8 (100%)		8/8 (100%)	
**Vitrification**	4/5 (80%)		5/5 (100%)	
**FCS/DMSO**	2/3 (67%)		7/7 (100%)	

Abbreviations: FCS = Fetal Calf Serum, DMSO = Dimethyl sulfoxide, F = generation number, PDX= patient-derived xenograft.

**Supplementary Table 3**. Preparation of solutions needed for vitrification and thawing.				
**Vitrification**	**Rinse medium**	**VS 1**	**VS 2**	**VS 3**
Temperature	Room temp	Room temp	Room temp	4°C
HBSS (mL)	18	16.5	15	12
FCS (mL)	2	2	2	2
DMSO (mL)	-	0.5	1	2
Propanediol (mL)	-	0.5	1	2
Ethylene Glycol (mL)	-	0.5	1	2
PVP (g)	-	-	-	2
Time (min)	5	5	10	10
**Thawing**	**TS 1**	**TS 2**	**TS 3**	**TS 4**
Temperature	Room temp	Room temp	Room temp	Room temp
HBSS (mL)	10	10	10	9
Sucrose (g)	1.71 (0.5M)	0.86 (0.25M)	0.43 (0.125M)	-
FCS (mL)	-	-	-	1
Time (min)	2	5	5	5
Abbreviations: VS= Vitrification solution, HBSS= Hank's balanced salt solution , FCS= Fetal Calf Serum, DMSO= Dimethylsulfoxide, PVP= Polyvinylpyrrolidone, TS= Thawing solution.				

Supplementary table 4. Antibodies used for immunohistochemical staining

Antigen	Antigen retrieval	Company (catalogue no.)	Dilution	Incubation time	Detection method
Ki67	TRIS/EDTA (pH 9.0)	DAKO (M7240)	1:350	60 minutes 20 ºC	RAMpo-GARpo
CD31	Citrate (pH 6.0)	Dianova (DIA 310)	1:100	60 minutes 20 ºC	RARbio-StrepHRP
WT1	Citrate (pH 6.0)	Dako (M3561)	1:100	60 minutes 20 ºC	RAMpo-GARpo
Vimentin anti human	EDTH (pH 8.0)	Santa Cruz (sc 6260)	1:150	60 minutes 20 ºC	RAMpo-GARpo
Vimentin anti human/mouse	Citrate (pH 6.0)	Cell signaling (D21H3)	1:50	o/n 4ºC	GARpo-RAGpo
ER	TRIS/HCl (pH 9.0)	Ventana (SP1)	NA	60 minutes 20 ºC	GARbio-StrepHRP
PR	TRIS/HCl (pH 9.0)	Ventana (1E2)	NA	60 minutes 20 ºC	GARbio-StrepHRP

Abbreviations: RAMpo= Rabbit- anti- Mouse horseradish peroxidase, GARpo= Goat- anti- Rabbit horseradish peroxidase, RAGpo= Rabbit-anti-Goat horseradish peroxidase, RARbio= Rabbit- anti- Rat biotinylated, GARbio= Goat- anti- Rabbit biotinylated, StrepHRP= streptavidin horseradish peroxidase, o/n = overnight, NA= non applicable.
